# Emerging Phage Resistance in *Pseudomonas aeruginosa* PAO1 Is Accompanied by an Enhanced Heterogeneity and Reduced Virulence

**DOI:** 10.3390/v13071332

**Published:** 2021-07-10

**Authors:** Pawel Markwitz, Tomasz Olszak, Grzegorz Gula, Magdalena Kowalska, Michal Arabski, Zuzanna Drulis-Kawa

**Affiliations:** 1 Department of Pathogen Biology and Immunology, University of Wroclaw, 51-148 Wroclaw, Poland; pawel.markwitz2@uwr.edu.pl (P.M.); tomasz.olszak@uwr.edu.pl (T.O.); grzegorz.gula@uwr.edu.pl (G.G.); 2 Division of Medical Biology, Institute of Biology, Jan Kochanowski University, 25-406 Kielce, Poland; magdalena.kowalska@ujk.edu.pl (M.K.); arabski@ujk.edu.pl (M.A.)

**Keywords:** *Pseudomonas aeruginosa*, biofilm, phage-resistant mutants, virulence, heterogeneity

## Abstract

Bacterial surface structures of a proteinic nature and glycoconjugates contribute to biofilm formation and provide shields to host defense mechanisms (e.g., the complement system and phagocytosis). A loss or alteration of these molecules, leading to phage resistance, could result in fewer virulent bacteria. In this study, we evaluate the biology and phenotype changes in *Pseudomonas aeruginosa* PAO1 phage-resistant clones, which emerge in phage-treated biofilms. We characterize these clones for phage-typing patterns, antibiotic resistance, biofilm formation, pathogenicity, and interactions with the innate immune system. Another important question that we address is whether phage-resistant mutants are also generated incidentally, despite the phage treatment-selective pressure, as the natural adaptation of the living biofilm population. It is found that the application of different phages targeting a particular receptor selects similar phage resistance patterns. Nevertheless, this results in a dramatic increase in the population heterogeneity, giving over a dozen phage-typing patterns, compared to one of the untreated PAO1 sessile forms. We also confirm the hypothesis that “phage-resistant bacteria are more susceptible to antibiotics and host-clearance mechanisms by the immune system”. These findings support phage application in therapy, although the overall statement that phage treatment selects the less virulent bacterial population should be further verified using a bigger collection of clinical strains.

## 1. Introduction

*Pseudomonas aeruginosa* is an aquatic-inhabiting bacterium and one of the main etiological factors of hospital- and ventilator-associated pneumonia, as well as chronic pulmonary infection in cystic fibrosis patients. Due to the involvement of a wide range of virulence factors, infections caused by these bacteria can pose a serious threat to the lives of patients, especially in the case of immunosuppression [1]. Among these factors, we can distinguish cellular membrane-related agents (type IV pili/T4P, flagella, LPS, and alginate) and various types of secretion systems and off-cell agents (exotoxins, elastase, phenazine dyes, and siderophores) [2]. This is particularly alarming, as *P. aeruginosa* is an “ESKAPE” group pathogen [3,4] and has many drug-resistance mechanisms, such as the formation of biofilm with an alginate barrier, drug alteration, the modification of drug-binding targets, changes in cell membrane permeability, and efflux pumps. The rapid increase of *P. aeruginosa* antibiotic resistance is especially problematic for cystic fibrosis patients due to chronic lung colonization [5,6]. *P. aeruginosa* is characterized by a huge clonal diversity, a fast mutation rate, providing an extensive genetic diversity, and fast adaptation to external conditions. This phenotypic and genotyping variation can be seen in the sessile population forming a biofilm [7]. As stated by Allison et al. [8], bacterial populations should be considered as a collection of multiple subpopulations, instead of a homogeneous culture, even when using a unique bacterial strain. In other words, the diversity of the *P. aeruginosa* population results from the phenotypic heterogeneity of the individual clones. Apart from metabolically active cells, the population also contains so-called “persister cells” as a backup, with a slowed-down metabolism, thus resisting both unfavorable environmental conditions and deliberately using antibacterial preparations (also phages) [9]. Recent studies have confirmed that the resistance to antibiotics or phages observed in the *P. aeruginosa* population does not always depend on genetic modification. A detailed analysis of individual clones reveals that selective pressure (phage or antibiotic) resulted in point mutations or large genomic deletions in the *galU* region only in exceptional cases [5,10]. Phages undeniably drive the evolution of each bacterial species and enhance the variability of *P. aeruginosa* as well. Phages—as obligatory bacterial parasites—are widely spread in host-related environments. They can be extracted at high concentrations from both soil and water. Their life cycle can follow several schemes: lytic, lysogenic, pseudolysogenic, and chronic infection. The lytic cycle is most desirable for industrial and medical purposes due to its bacterial eradication capabilities. Generally, phages are highly specific in host receptor recognition. This makes it potentially useful for therapy, food decontamination, medical device disinfection, and bacterial detection. It is worth remembering that phages are an integral part of the environment and affect the bacterial population in a very complex way. Particularly important is the involvement of phages in horizontal gene transfer, which is responsible for the biodiversity of both bacterial viruses and their hosts [11]. Phage therapy never leads to the complete eradication of pathogens due to the emergence of phage-resistant mutants and the existence of so-called “persistent cells” within the bacterial population. Nevertheless, the administration of bacteriophages supports the antibacterial action of the immune system. The latest in vivo studies indicate that the development of phage-resistant mutants does not affect phage therapy effectiveness in immunocompetent patients [12,13]. The antagonistic coevolution of the phage–host relationship forces adaptation changes both in bacteria and viruses. Bacterial mechanisms preventing viral infection may go in many different ways. The most common strategy is the inhibition of phage adsorption to the cell surface by blocking access, changing the structure, or deleting the phage target receptors. These changes can affect some substantial cell functions (e.g., biofilm formation, surface adhesion, motility, or antibiotic resistance). Moreover, recently gained resistance usually protects against other phages recognizing the same receptor [14,15]. On the other hand, a loss or alteration of cell wall molecules, leading to phage resistance, makes bacteria defenseless against the immune system (e.g., serum complement system or phagocytosis) [16,17]. Therefore, the bacterial population has to cope somehow with the viral infection and sensitization to the immune response at the same time.

In the present study, we analyze the phenotype and virulence properties of the PAO1 biofilm population infected with a single phage or phage cocktail, and we determine how virus selection affects the bacterial heterogeneity and whether those modifications make the emerging population less virulent and more sensitive to the host defense system. 

## 2. Materials and Methods

### 2.1. Experimental Pipeline 

**Figure 1:**
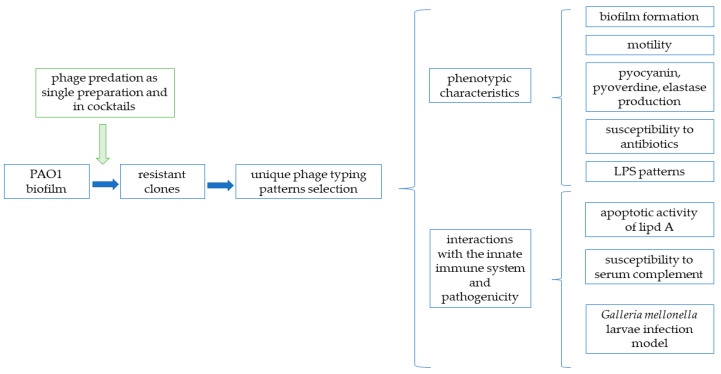
The scheme of the experimental pipeline and methodology used to determine the emergence of phage resistance in a *P. aeruginosa* PAO1 biofilm population and its impact on bacterial heterogeneity and virulence.

Figure 1 presents the overall procedures and methodology approaches utilized in this study. 

### 2.2. Bacterial Strains and Bacteriophages

The Pseudomonas aeruginosa PAO1 (ATCC 15692) reference strain was used. The bacteria were stored at −70 °C in Trypticase Soy Broth (TSB, Becton Dickinson and Company, Cockeysville, MD, USA), supplemented with 20% glycerol. The collection of seven lytic Pseudomonas phages listed in Table 1 were applied as the selective agents.

### 2.3. Isolation of Phage-Resistant Clones

The bacterial biofilm was infected either by individual phage (KT28, KTN6, LUZ7, LUZ19, phiKZ, and KTN4) or phage cocktails composed of two (KTN6 + LUZ7, KTN6 + phiKZ, KT28 + phiKZ, and KTN6 + KTN4) or three phages (KTN6 + LUZ7 + phiKZ and KTN6 + LUZ7 + KTN4). The cocktails were designed based on the phage biology and recognized receptors (Table 1). The surviving bacterial clones were isolated from the biofilm population after phage infection.

An bacterial culture grown overnight in Trypticase Soy Broth (TSB, Oxoid, Basingstoke, UK) was diluted to 1 × 10^5^ CFU/mL and transferred to a 96-well plate (Nunc, Roskilde, Denmark), covered with a peg-lid and incubated at 37 °C for 24, 48, or 72 h. The peg-lid cover with established biofilms was then transferred to a new plate with a TSB medium containing phages (or phage cocktails) at 10^7^ PFU/mL titer and incubated for 24 h at 37 °C. Next, the peg-lid was rinsed in phosphate-buffered saline (PBS; 1 mM KH_2_PO_4_, 3 mM Na_2_HPO_4_-7H_2_O, 156 mM NaCl, pH 4) buffer to wash out the remaining planktonic forms, and the biofilm fraction was collected using an ultrasonic bath and immediately plated for the colony count on a Trypticase Soy Agar medium (TSA, Oxoid, Basingstoke, UK). From each variant of the experiment, around 10 discrete colonies were randomly isolated, which gave a total of 417 different clones (including 30 control clones not treated with phages) (Table S1).

### 2.4. Phage Typing by the Double-Layer Plate Method

The bacterial culture grown overnight was transferred to melted soft agar tubes (TSB, 0.5% technical agar, Oxoid, Basingstoke, UK) and poured into TSA plates. All examined phages were diluted in PBS buffer to 10^5^ PFU/mL and spotted (10 µL) on previously prepared plates of bacterial lawns. The plates were incubated for 24 h at 37 °C, and the observed plaques showed the sensitivity of an isolate to a given phage. Phage-typing was repeated at least three times and inspected visually. The difference in phage patterns allowed for the selection of isolates for detailed phenotype analysis.

### 2.5. Motility Assays

The bacterial culture grown overnight was diluted to 10^5^ CFU/mL and transferred to Petri dishes containing a TSA medium with different concentrations of agar (0.3% for swimming motility, 0.8% for swarming motility, and 2% for twitching motility). Using the pipette tip, the bacterial suspension was transferred by a puncture into the agar medium (at 1/2 depth for swimming and swarming motility and at full depth for twitching motility). The plates were then incubated at 37 °C for 24 h (swimming and swarming motility) and 48 h (twitching motility). After the incubation, the growth zones were measured. For swimming and swarming motility, the measurement was made directly, while for twitching motility—after removing the agar layer and staining the bottom of the plate with a 0.01% solution of crystal violet (CV, Sigma-Aldrich, St. Louis, MO, USA). Each variant of the experiment was performed in at least 5 repetitions [18,19,20]. For statistical analysis, the one-way ANOVA (the Tukey test) for independent groups was used (*p*-values < 0.001 were regarded as significant).

### 2.6. Pyocyanin and Pyoverdine Production

The bacterial culture grown overnight was diluted to 1 × 10^5^ CFU/mL in a TSB medium, transferred to a 24-well plate (Sarstedt, Nümbrecht, Germany), and incubated at 37 °C for 48 h. After incubation, each bacterial suspension was collected in an Eppendorf tube and centrifuged at 10,000 RPM. The obtained supernatants were transferred to a transparent 96-well plate (for pyocyanin measurement) and a black 96-well plate (for pyoverdine measurement). The absorbance for pyocyanin was measured using a Varioskan Lux microplate reader (Thermo Fisher Scientific Inc., Waltham, MA, USA) at λ = 686 nm, and the fluorescence for pyoverdine at λ = 395 nm (excitation) and λ = 460 nm (emission). Five repetitions were made for each experiment option [21]. For the statistical analysis, the one-way ANOVA (the Tukey test) for independent groups was used (*p*-values < 0.001 were regarded as significant).

### 2.7. Elastase Production

The bacterial culture grown overnight was diluted to 1 × 10^5^ CFU/mL in a TSB medium, transferred to a 24-well plate, and incubated at 37 °C for 48 h. After incubation, each bacterial suspension was collected in an Eppendorf tube and centrifuged at 10,000 RPM for 10 min. The supernatants were then mixed with 900 μL of 100 mM Tris and 1 mM CaCl_2_ buffer (pH 7.5) and 5 mg of ECR (Elastin Congo Red, Sigma-Aldrich, St. Louis, MO, USA). Then, the samples were incubated at 37 °C for 3 h with agitation. The reaction was stopped by placing the probes in ice and adding 1 mL of 0.7 M phosphate buffer (pH 6.0). Finally, the tubes were centrifuged at 10,000 RPM for 10 min, and the absorbance was measured at λ = 495 nm with a Varioskan Lux microplate reader (Thermo Fisher Scientific Inc, Waltham, MA, USA). Five replicates of each experiment were performed [22]. For the statistical analysis, the one-way ANOVA (the Tukey test) for independent groups was used (*p*-values < 0.001 were regarded as significant).

### 2.8. Antibiotics Susceptibility Testing

The antibiotic susceptibility of the PAO1 host strain and phage-induced mutants was evaluated with the E-test method to determine the minimum inhibitory concentrations (MICs), according to the European Committee on Antimicrobial Susceptibility Testing (EUCAST) recommendations (https://www.eucast.org/ (accessed on 1 January 2019); EUCAST breakpoint tables, Version 9.0), with the application of gentamicin (MIC Strip GEN 0.5–32 mg/L), piperacillin (MIC Strips PIP 2–128 mg/L), ceftazidime (MIC Strips CAZ 0.5–32 mg/L), ciprofloxacin (MIC Strips CIP 0.06–4 mg/L) (Liofilchem, Roseto degli Abruzzi, Italy), and imipenem (ETEST^®^ Imipenem IP32, BioMerieux SA, Chemin De L’Orme, Marcy L’Etoile, France).

### 2.9. Biofilm Formation Measurement

The bacterial culture grown overnight was diluted to 10^5^ CFU/mL in a TSB medium, transferred into the 96-well plate (Nunc, Roskilde, Denmark), covered with a peg-lid, and incubated at 37 °C for 48 h. After incubation, the peg-lid plates were rinsed with the PBS buffer and stained with 0.01% CV. The dye was then eluted with 96% ethanol, and the absorbance value was measured at λ = 560 nm with a Varioskan Lux microplate reader (Thermo Fisher Scientific Inc., Waltham, MA, USA) [23,24]. For the statistical analysis, the one-way ANOVA (the Tukey test) for independent groups was used (*p*-values < 0.001 were regarded as significant).

### 2.10. Susceptibility of Bacterial Isolates to Serum Complement Activity

The liquid culture of examined bacterial strains kept overnight was diluted to 10^6^ CFU/mL and added to an equal volume of active or heat-inactivated (56 °C, 30 min) sheep serum (ProAnimali, Wroclaw, Poland). The prepared suspensions were incubated for 2 h at 37 °C. The colony counts CFU/mL were measured every 30 min by serially plating diluted samples on a TSA medium. The experiments were performed at least in triplicate. The results in the active serum were interpreted as follows: (i) resistance to serum complement as a 1 log increase in the final CFU/mL; (ii) moderate sensitivity as a 0–1 log decrease in the final CFU/mL; (iii) sensitivity as a >1 log decrease in the final CFU/mL.

### 2.11. Lipopolysaccharide Isolation and Pattern Analysis

An examination of LPS structure pattern was performed using the slightly modified Marolda of the classical Westphal and Jann protocol, utilizing the hot aqueous phenol extraction [25,26]. The bacterial cultures grown overnight in the TSB medium (Oxoid, Basingstoke, UK) were centrifuged and adjusted to OD600 equal to 2.0 in the PBS buffer. The bacterial cells were disintegrated with lysis buffer (2% SDS, 4% β-mercaptoethanol, Tris, pH 6.8, 1000 °C, 15 min), and the proteins were enzymatically digested with proteinase K (20 mg/mL, 600 °C, 1 h). The protein debris was eliminated, and the LPS was isolated by a hot aqueous phenol solution (90%, 70 °C, 15 min.). The LPS containing aqueous phase was purified using ethyl ether to remove residual phenol. To assess the concentration of LPS used for electrophoresis, the Purpald method of KDO (3-Deoxy-D-manno-oct-2-ulosonic acid) measurement was performed [27]. Subsequently, the LPS samples were separated by the Tricine-SDS-PAGE method (14% polyacrylamide gel with 4 M urea, 80 V constant) [26]. Finally, after separation, the LPS bands were detected by silver staining [28].

### 2.12. Apoptotic Activity of the LPS Isolated from Phage-Resistant PAO1 Mutants

The proapoptotic properties of LPSs were determined using the Annexin V-FITC apoptosis detection Kit I (BD Pharmingen, Franklin Lakes, NJ, USA) and Cleaved PARP FITC MAB Detection Kit (Becton Dickinson, Franklin Lakes, NJ, USA) against normal human bronchial epithelium BEAS-2B cells (ATCC^®^ CRL-9609™). Phosphatidylserine is used to detect apoptotic cells, because as it is translocated to the outer cytoplasmic membrane during apoptosis, it interacts with annexin in the presence of calcium ions [29]. The BEAS-2B line was used as a model of eukaryotic cells, because it exhibited the highest homology in terms of the gene expression pattern, with primary cells and the lowest number of dysregulated genes, compared with non-tumoral lung tissues (comparison of the expression profiles of 380 genes encoding proteins involved in the metabolism of xenobiotics in 10 commonly used lung cell lines and four primary cultures of human bronchial epithelial cells) [30]. The BEAS-2B cells were treated with *P. aeruginosa* PAO1 LPSs isolated from phage-resistant mutants or lipid A *Burkholderia cepacia* (as a standard) at a concentration range of 10–500 ng/mL for 48 h. BEAS-2B cells were cultured in an LHC9 medium (Invitrogen, Waltham, MA, USA) at 37 °C. Both cell lines were cultured in a humidified 5% CO_2_ atmosphere.

The first step of this part of the study was focused on the selection of proper tests for the analysis of the proapoptotic properties of LPS. Annexin V-FITC apoptosis detection is a standard technique for the analysis of the apoptosis of chemical compounds, but its sensitivity for LPS study could be verified. Annexin might interact with LPS by lipid A, depending on the calcium ions. The lack of calcium ions decreases the interaction of annexin with phosphatidylserine (protein used to detect apoptotic cells) [29]. The lipid A of *B. cepacia* was used to verify the Annexin V-FITC apoptosis detection Kit I and Cleaved PARP FITC MAB Detection Kit. Based on the obtained results, the second one was chosen for the analysis of the apoptotic activity of the LPS isolated from phage-resistant *P. aeruginosa* PAO1 mutants (Figure S1).

*Annexin V-FITC apoptosis detection Kit I*. BEAS-2B cells were washed twice with cold PBS and then resuspended in 1 × binding buffer at a concentration of 1 × 10^6^ cells/mL. An aliquot of 125 µL of the cell suspension was incubated with 5 µL of annexin V-FITC and 5 µL of propidium iodide (PI) at room temperature for 15 min in the dark. The cells were resuspended in 400 µL of 1 × binding buffer. The fluorescence was determined using a Becton Dickinson LSR II flow cytometer. A computer software (BD FACS DiVa, version 6.1.3, Becton Dickinson) was used for the data acquisition and analysis. The data for 20,000 events were stored. A cell gate containing BEAS-2B was established based on a forward and side light scatter. Four different populations of cells were detected with the Annexin V-FITC kit: normal cells that were Annexin-negative and PI-negative and expressed no fluorescence, early apoptotic cells that were Annexin-positive and PI-negative and expressed a green fluorescence, late apoptotic/necrotic cells that were Annexin-positive and PI-positive and expressed a green and orange fluorescence, and necrotic cells that were Annexin-negative and PI-positive and expressed an orange fluorescence. All the samples were measured in three independent experiments [31]. Additionally, BEAS-2B cells after 15 passages (naturally “coming” in the apoptosis pathway) were used to test the sensitivity of this method for the analysis of proapoptotic properties of LPS, taking into account the possibility of annexin–LPS interaction by lipid A [29].

*Cleaved PARP FITC MAB Detection Kit*. BEAS-2B cells at exponential growth were incubated with LPSs at 10–100–500 ng/mL. Briefly, the cells were washed twice in cold PBS and resuspended in a BD Cytofix/Cytoperm™ solution for the permeabilization of the nuclear membrane at a concentration of 1 × 10^6^ cells/mL (20 min on ice). The cells were washed twice, resuspended in BD Perm/Wash™ buffer, and incubated with the antibody at room temperature for 30 min in the dark. The fluorescence of the PAPR-1 protein was determined using an LSR II flow cytometer (Becton Dickinson, Franklin Lakes, NJ, USA). A computer software, BD FACS DiVa (version 6.1.3, Becton Dickinson), was used for the data acquisition and analysis, and 20,000 cells per point were analyzed for PARP-1 fluorescence intensity. All samples were measured in three independent experiments. Camptothecin was used as the positive control. For the statistical analysis, the one-way ANOVA (the Tukey test) for independent groups was used (*p*-values < 0.05 were regarded as significant).

### 2.13. Galleria mellonella Larvae Infection Model

The *G. mellonella* model was used to determine the virulence of the investigated PAO1 clones. Before the experiments, the larvae were selected in terms of size (approx. 20 mm) and acclimatized for a week at 15 °C. Then, the overnight bacterial cultures were centrifuged (10 min/10,000 RPM), and the sediment was suspended in PBS buffer. The prepared suspensions were diluted to 10^3^ CFU/mL and injected into the hindmost proleg of wax moth larvae (10µL~10 CFU/larvae). The infected larvae were incubated for 72 h at 37 °C, and their viability was controlled after 8, 18, 24, 48, and 72 h. For each bacterial clone, the experiment was performed on 20 larvae. The positive control consisted of larvae infected with a wild-type strain of PAO1, while in the negative control, the larvae were injected with PBS buffer [32,33]. The results were visually examined, and the results were statistically curated using GraphPad Prism 6.0 (GraphPad Software Inc., La Jolla, CA, USA). For the statistical analysis, the log-rank Mantel-Cox test was used (*p*-values < 0.05 were regarded as significant).

## 3. Results

### 3.1. Heterogeneity in Phenotypic Patterns

**Figure 2:**
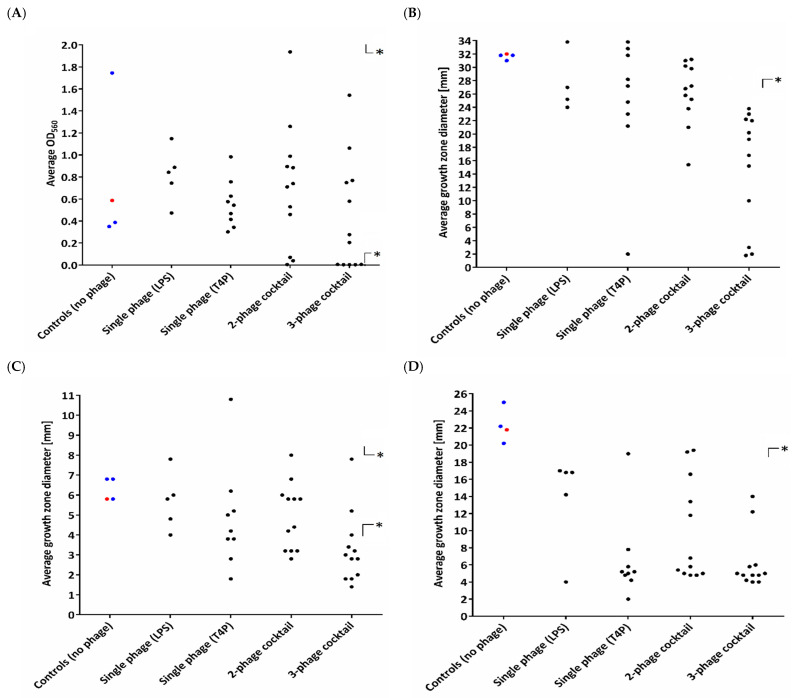
Changes in the biofilm formation ability and motility factors in the emerging phage-resistant PAO1 clones versus wild-type PAO1 sessile population: (**A**) biofilm formation; (**B**) swimming; (**C**) swarming; (**D**) twitching. Untreated sessile PAO1 was used as a control. The dots represent the measured values of the particular clones: wild-type clones (red dot); control sessile clones (blue dots); selected mutant phages (black dots). Statistically significant difference, compared to the phage-untreated pool (* *p* < 0.001).

Based on the phage-typing results of 417 randomly isolated biofilm clones (untreated control and phage-treated) (Table S1), it was found that PAO1 infected with different phages targeting a particular receptor display a similar phage resistance arrangement when typed with the collection of seven lytic phages. Nevertheless, the diversity of phage-typing patterns varied between 4–10 for clones selected by single-phage treatment, whereas the number of types reached 14–17 after the application of the cocktails. The variety among the phage-selected clones only differed from the one phage-typing pattern observed for untreated clones isolated from the biofilm. At this step of the study, we were able to conclude that phage infection drives a huge heterogeneity within the PAO1 population, and it was not related to a specific sessile style of life. Taking into account the aforementioned results, the pool of clones dedicated for further biology and phenotyping analyses has been narrowed to 42 clones in total. Four groups of clones were then created: (i) treated with LPS-dependent phages (5 clones); (ii) treated with T4P-dependent phages (9 clones); (iii) treated with a double-phage cocktail (12 clones); (iv) treated with a triple-phage cocktail (12 clones). Clones were selected in terms of the widest possible variety in phage-typing and colony morphology. As the control, three sessile representatives and a wild type were taken. The clonal heterogeneity of the phenotypic properties, apart from phage-typing, was also analyzed in terms of biofilm production, motility, colony growth features, and susceptibility to antibiotics (Figure 2, Table S2). The biofilm formation experiments revealed that only clones treated with phage cocktails showed statistically significant changes (Figure 2A), and the values of the average biofilm amount were widely distributed within both the control representatives and phage-resistant isolates, regardless of the applied phage preparation. The modification of colony features, such as brown or grey pigmentation and mucoid version, was correlated with the selection of phage cocktail, and a large deletion in the *galU* region of the genome often occurred (Table S2) [2].

In terms of the *motility properties* (Figure 2B*–*D), the untreated control isolates presented a relatively consistent ability to swim, swarm, and twitch, whereas for the phage-resistant clones, the motility results were widely distributed. Nevertheless, the emergence of resistance to phages, regardless of the group, was unequivocally correlated with a dramatic loss of twitching, and 27/38 phage-resistant clones exhibited a statistically significant decreased swimming ability, combined with reduced swarming. The highest impact on the PAO1 motility was seen after the application of a triple-phage cocktail. A similar conclusion was drawn regarding the antibiotic susceptibility tests, as most of the alterations exceeding a fourfold MIC reduction considered as significant were presented by isolates after a triple-phage cocktail treatment. In general, the increased susceptibility was mostly attributed to gentamicin in the clones treated with both double- or triple-phage cocktails. Some clones had reduced MICs of piperacillin, ciprofloxacin, and imipenem. All the mutants showing a major change in antibiotic susceptibility belonged to the so-called “brown phenotype”, which was associated with a large deletion in the galU region (Table S2) [2].

### 3.2. Diversity in Extracellular Virulence Factor Production

**Figure 3:**
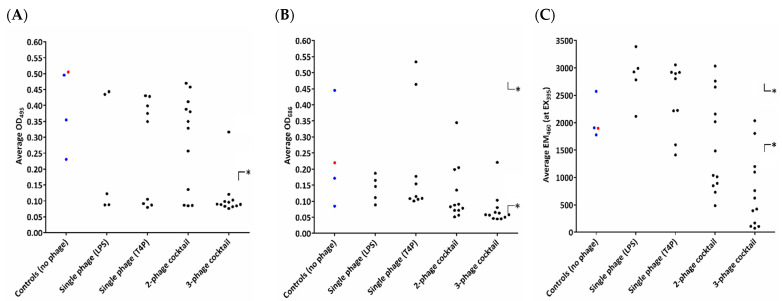
Changes in extracellular virulence factor production in emerging phage-resistant PAO1 clones versus the wild-type PAO1 sessile population: (**A**) changes of elastase in the growth medium; (**B**) changes of pyocyanin in the growth medium; and (**C**) changes of pyoverdin in the growth medium. Untreated sessile PAO1 was used as a control. The dots represent the measured values of particular clones: wild-type PAO1 clones (red dot); control sessile clones (blue dots); and mutants selected by phages (black dots). Statistically significant difference compared to the phage-untreated pool (* *p* < 0.001, ANOVA test).

As the flexibility of *P. aeruginosa* to colonize different environments is largely dependent on secretory virulence factors, we examined three, which are arguably the most important (elastase, pyocyanin, and pyoverdine) (Figure 3). Regardless of the phage used during the experiments, most of the tested isolates (23/38) showed a statistically significant decrease in elastase production. The measurements of the intensity of pyocyanin and pyoverdine production were no longer as conclusive as in the case of elastase. The production of pyocyanin was decreased in 1 isolate (Table S2, clone #6) treated with LPS-dependent phages and 18 clones after the application of cocktails. On the other hand, T4P-dependent phages selected resistant clones, with an induced production of pyocyanin. The highest variety in extracellular virulence factor production in PAO1 was recorded for pyoverdine. The overproduction was strongly selected by single phages, but this impact was diminished with the cocktail combination, where 17/24 were characterized with a reduced pyoverdine production.

### 3.3. Interactions with the Innate Immune System and Pathogenicity

#### 3.3.1. Phage Resistance Does Not Impact the Apoptotic Activity of LPS

**Figure 4:**
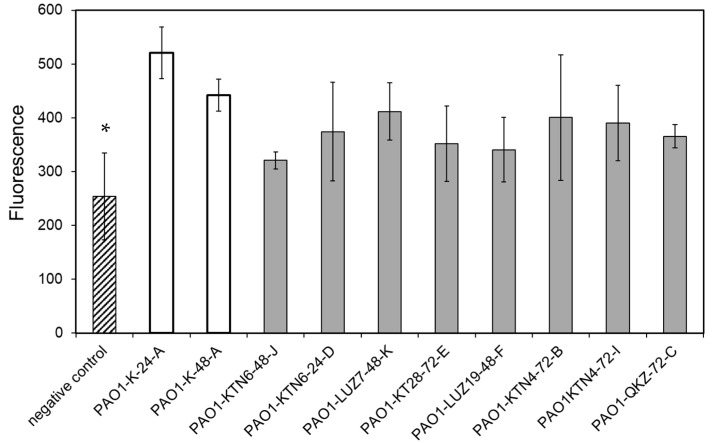
The apoptotic properties of LPSs at 100 ng/mL, isolated from phage-resistant *P. aeruginosa* PAO1 mutants and measured after 48 h by the Cleaved PARP FITC MAB Detection Kit. The negative control consisted of untreated BEAS-2B cells; white bars—LPS from phage-untreated PAO1 clones; grey bars—LPS from phage-selected PAO1 clones. Statistically significant difference compared to the LPS from the phage-untreated pool (* *p*-values < 0.05, ANOVA test).

Lipid A, as a part of LPS, might disrupt this mechanism of apoptosis detection via this lipoglycan interaction with calcium ions. Figure S1A shows that in BEAS-2B, after 15 passages (physiological decrease of apoptotic cells in the control), the percentage of early/late apoptotic and necrotic cells in the control is higher after incubation with lipid A. This indicates that lipid A might block the detection of apoptosis (Figure S1B). Moreover, the sensitivity of the Cleaved PARP FITC MAB Detection Kit for the lipid A study (Figure S1C) is much higher than the Annexin V-FITC apoptosis detection Kit I (Figure S1A, cells at exponential growth in 10 passages). The Cleaved PARP FITC MAB Detection Kit was chosen to measure the apoptotic activity of LPS isolated from phage-resistant *P. aeruginosa* PAO1 mutants at a range of 10–100–500 ng/mL (Figure S2). The statistically significant differences have been obtained for 100 ng/mL, and this concentration was used to test the apoptotic activity of LPS derived from selected phage-resistant mutants. It turned out that there was no statistically significant difference between the activity of LPS isolated from phage-sensitive versus phage-resistant PAOl clones, and all of the samples induced apoptosis in BEAS-2B cells after 48 h (Figure 4). This may suggest that lipid A remained unmodified during the emergence of phage resistance.

#### 3.3.2. LPS-Dependent Phage Selection Sensitizes Bacteria to Serum Complement

**Figure 5:**
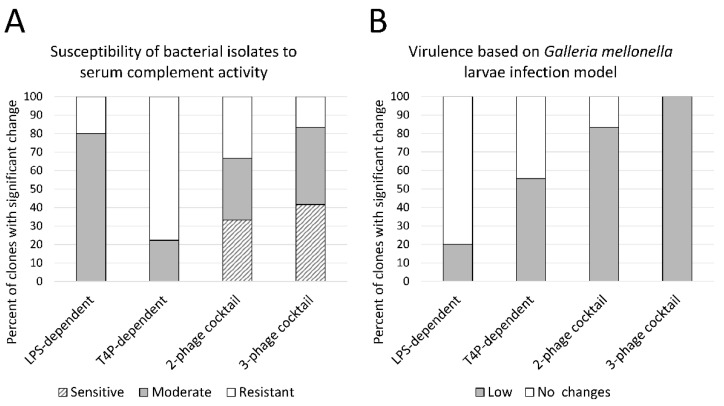
Changes in the virulence in emerging phage-resistant PAO1 clones versus the wild-type PAO1 sessile population measured as: (**A**) susceptibility to serum complement; white bars—resistance to serum complement (1 log increase in the final CFU/mL); light grey bars—moderate sensitivity (0–1 log decrease in the final CFU/mL); striped bars—sensitivity (>1 log decrease in the final CFU/mL); (**B**) survival rate in the *G. mellonella* infection model; white bars—no changes; grey bars—a significant decrease (*p* < 0.05). Untreated sessile PAO1 were used as a control. The statistical analysis for the *G. mellonella* model was conducted using the log-rank Mantel-Cox test.

The second aspect of the LPS function towards the immune system is the oligosaccharide chain (O antigen) important, for instance, in the protection against serum complement activity. Therefore, an LPS pattern analysis was next performed and visualized on SDS-PAGE-Tricine gel (Figure S3, Table S2). Rough (R) or semi-rough (SR) forms of LPS were observed in the clones treated with LPS-targeting phages, which was expected as the primary receptor modification strategy used by the infected bacterial population. We were able to distinguish different patterns of R-LPS or SR-LPS patterns (Figure S3). The susceptibility of the bacterial isolates to serum complement activity was analyzed in comparison to the LPS profiling, as severe modifications to it usually result in a sensitization to the complement cascade (Figure 5, Table S2, Figure S3). Indeed, the application of LPS-dependent phages impaired both the LPS pattern and the susceptibility to serum lytic activity, regardless of whether a single or phage mixture treatment was employed. The serum resistance did not change much among the clones selected by T4P-dependent phages (only 2 clones with a moderate susceptibility), with no LPS structural modifications. In the pool treated with a phage cocktail, 17/24 isolates exhibited a reduced CFU/mL in the active serum, which was accompanied by the LPS-modified patterns.

#### 3.3.3. Phage Cocktail Application Results with a Significantly Reduced Virulence in the *G. mellonella* Infection Model

The final experiments were dedicated to the verification of the impact of phage resistance on bacterial virulence in the wax moth larvae infection model (Figure 5). In the group of isolates treated with LPS-dependent phages, only one clone among the 5 tested clones displayed a reduced virulence in contrast to the 5/9 treated with T4P-dependent phages. The best disarming effect was obtained for the PAO1 treatment with phage cocktails, as about 90% of phage-resistant clones turned out to kill a significantly lower number of the infected *G. mellonella* larvae. This could be expected, as in this group of clones, most of the tested virulence agents (motility, extracellular factors production, and serum resistance) were reduced. Nevertheless, only the combination of different attenuated factors finally gave the overall lower pathogenicity in the in vivo model.

## 4. Discussion

The co-evolution of phages and their hosts has been the subject of intense medical and environmental research, particularly over the past decade. So far, it has been established that phage pressure has an indisputable effect on the biodiversity of bacterial populations [5,8]. Even within a narrow field of microbiological interest, namely, the effect of phages on the *P. aeruginosa* genetics, the literature is reporting cross-resistance events, receptor modification and the occurrence of a large deletion [1,6,19,20,21]. Several studies are also describing the anti-pseudomonads activity of lytic phages as a single and cocktail preparation against both planktonic and biofilm populations [34,35,36]. It has been stated that there are no synergistic or antagonistic effects among phages when combining them from the different taxonomic groups or targeting different receptors. The utilization of phage cocktails was considered superior, especially for biofilm eradication.

Considering the above, our study is focused on describing the changes in the *P. aeruginosa* PAO1 biology associated with the alteration of key virulence factors in response to phage predation events. The second aim was to determine the relationship between the emergence of phage resistance and modifications in the heterogeneity of the examined population. To accomplish this, we first compared the results of the phage-typing of 30 *P. aeruginosa* control isolates not treated with phages to a total of 387 isolates selected by a single phage or phage cocktail. For in-depth examination, a group of isolates containing all the major phage-typing patterns and an abnormal colony morphology was selected for phenotypic and virulence characteristics analyses.

In our study, the control populations (irrespective of whether they contained wild-type strains or biofilm isolates) displayed a homogeneous susceptibility to the phage panel used for typing. In contrast, we detected a wide variation of phage resistance patterns among the clones treated with a single phage or phage cocktails. Moreover, in each variant of the experiment, part of the population retained the phage profile of the wild-type strain. Therefore, it can be confirmed that the *P. aeruginosa* population infected with phages increases the clonal heterogeneity, creating clones with different phage sensitivity patterns, including cross-resistance to phages recognizing the same surface receptor. This observation is supported by other publications focusing both on populations with an altered phage resistance and on so-called “persister” cells, which are thought to be the source of the unchanged portion of the population [2,9,12,37,38,39,40].

To better understand the nature of the changes within the population under study, a series of experiments were carried out, targeting the expression of selected virulence factors in particular structural ones recognized by phages, as well as indicators of general bacterial fitness. The isolates were divided into groups based on the phage type or the number of phages in the cocktail they were treated with to observe the differences in the infection impact. The O-chain modifications in LPS were, as expected, only observed in clonal groups treated with LPS-dependent phages. Surprisingly, there was a decrease in all motility types in each study group. This was particularly evident in the case of twitching, which was decreased in every tested clone. Similar results were reported by Pires et al., 2017 [2] and Hosseinidoust et al., 2013 [41]. Twitching, as a flagellar-independent type of motility, is associated with T4P [42,43]. This may suggest that the reduction of mobility is some kind of natural phenotypical reaction of the bacterial population to the appearance of phages in the environment.

Unlike the factors mentioned above, the bacterial biofilm is a much more complex structure, with many more genetic mechanisms involved in its production. The results presented in this publication indicate that the use of single phages has little effect on the ability of bacterial mutants to form a biofilm. Only a rapid response to the appearance of two or more phages resulted in an impairment of the bacterial population in this regard. Interestingly, a severe disruption of biofilm development is strongly associated with the presence of large genomic deletions within the *galU* gene vicinity, which generally reduce the metabolic activity of *P. aeruginosa* (depending, of course, on the size of the deletion) [2]. In general, we noticed a huge variation in the biofilm production ability within both control representatives and phage-resistant isolates, regardless of the applied phage preparation.

Secretory virulence factors, such as elastase, pyocyanin, and pyoverdine significantly facilitate the *P. aeruginosa* colonization of new niches but are not directly related to the cell response to phage infection. It appears, however, that under phage pressure, the bacterial population shows a highly variable intensity of production of these factors, and certain trends can be observed here. The most notable tendency, as it occurred in isolates across all experimental groups, was a reduction in elastase secretion. A similar effect was also observed for pyocyanin, but the noticeable decreases in the production of this dye were mainly found in isolates treated with phage cocktails. Somewhat unexpected results came from studying the level of pyoverdine production. Isolates treated with single phages (LPS- or T4P-dependent) were mostly characterized by an intensification of pyoverdine production, in contrast to the application of cocktails, where most phage-resistant clones showed a decrease in the production of this siderophore. In conclusion, while isolates treated with single phages showed quite a high variability in the production of secretory virulence factors, the selected isolates with cocktails (especially triple-phage cocktails) showed a decrease in the production of all three factors. Of course, a phenotypic variability was also seen among clones with large genomic deletions in the region for phenazine biosynthesis [2,10,12,37,38,39]. Phage infection is a major challenge for bacterial cells and affects their entire metabolism and therefore virulence. The level of virulence is difficult to determine unambiguously, as it is made up of many factors. We decided to evaluate the impact of emerging phage resistance on the key elements responsible for the interactions with the innate immune system, such as the sensitivity to the complement system, the apoptotic properties of LPS lipid A, and the pathogenicity in the *G. mellonella* larvae infection model.

Our study on the apoptotic properties of LPS lipid A indicated no significant difference between phage-sensitive versus -resistant bacteria in this regard, which may suggest that lipid A remained unmodified in emerging phage-resistant variants. In contrast, analyzing the sensitivity to complement cascade, it turned out that the wild-type PAO1 strain was fully resistant to serum bactericidal activity, unlike a large portion of mutants isolated after phage infection. Our results confirmed a previously observed correlation between loss of the O-chain type B LPS and sensitization to complement [44,45]. Serum-sensitive isolates occurred overwhelmingly in the group treated with LPS-dependent phages. In those cases, we detected variations in LPS patterns (semi-rough or rough). Among the PAO1 mutants, after T4P-dependent phage infection, only two showed a decrease in pyoverdine production and a concomitant increase in serum sensitivity and pyocyanin production. The aforementioned examples indicate that the bacterial response to a single phage infection might drastically differ from the consequences of a cocktail application.

The results of *G. mellonella* larvae infection assay indicate a reduced pathogenicity of isolates treated with T4P-dependent giant phages (KTN4 and phiKZ). This phenomenon was accompanied by the persistence of phages in the population and whatever the mechanism of maintaining the phage in the population (most likely pseudolysogeny) [37,40], and its presence caused a severe impairment of bacterial virulence and fitness in our study. It should also be mentioned that phage-resistant clones displaying no genetic modification may exhibit differences in phage susceptibility patterns, which may partly depend on physiological adaptation, rather than genetic variation. Similar conclusions have been drawn from analyses of the effects of certain antibiotics on bacterial genetics [10].

Summarizing the outcome of this study, we can say that the trend in *P. aeruginosa* PAO1 biology changes is related to the number of phages that cause selection pressure on the population. The more phages appear in the environment, the deeper and more noticeable are the phenotypic changes involving a reduction of various virulence factors’ production levels. The use of a phage mixture targeting different extracellular structures as receptors causes a huge impact on bacterial fitness, virulence, and pathogenicity. To draw a general assumption that phage predation disarms bacteria, a similar study should be undertaken on a big collection of *P. aeruginosa* strains. Nevertheless, our research indicates that the escape from subsequent phage infection leads to a strong diversification and heterogeneity of the PAO1 population.

## Figures and Tables

**Table 1:** Detailed characteristics of the bacteriophages used in this work.

Phage	Taxonomy (Family, Genus)	Genome Size	GenBank Accession Number	Recognized Bacterial Receptor
LUZ7 *	*Schitoviridae,* *Luzseptimavirus*	74,901 bp	NC_013691	LPS
KTN6 **	*Myoviridae*, *Pbunavirus*	65,994 bp	KP340288	LPS
KT28 **	*Myoviridae*, *Pbunavirus*	66,381 bp	KP340287	LPS
LUZ19 *	*Autographiviridae*, *Phikmvvirus*	43,548 bp	NC_010326	T4P
phiKZ *	*Myoviridae*, *Phikzvirus*	280,334 bp	AF399011	T4P
KTN4 **	*Myoviridae, Phikzvirus*	279,593 bp	KU521356	T4P
PA5oct **	*Myoviridae,* unclassified	286,783 bp	MK797984	LPS/T4P

* Laboratory of Gene Technology, KU Leuven, Leuven, Belgium. ** Department of Pathogen Biology and Immunology, University of Wroclaw, Wroclaw, Poland.

## Data Availability

Not applicable.

## Supplementary Materials

The following are available online at https://www.mdpi.com/article/10.3390/v13071332/s1. Table S1. Phage-typing patterns of biofilm-isolated *P. aeruginosa* PAO1 clones; Table S2. Characteristics of *P. aeruginosa* PAO1 control and phage-resistant isolates; Figure S1. The apoptotic assay measured by the Annexin V-FITC apoptosis detection Kit I, using *B. cepacia* lipid A as a standard (A), and Cleaved PARP FITC MAB Detection Kit (C). The possible role of lipid A (yellow) in apoptosis detection via annexin (green star) interactions with phosphatidylserine (black dots in the cell membrane), depending on calcium ions (red dots) (B). Figure S2. The apoptotic properties of *P. aeruginosa* PAO1 LPS isolated from two clones from the phage-noninfected biofilm. The BEAS-2B cells were treated with LPS at a range of 10–500 ng/mL, and the apoptotic effect was measured by the Cleaved PARP FITC MAB Detection Kit (C). * *p* < 0.05 (ANOVA test); Figure S3. LPS profiles of PAO1 clones isolated after controlled infection by selected phages analyzed in 14% polyacrylamide/tricine-SDS gels: (A) smooth profile; (B–C) semi-rough profile; (D–G) rough profile; Figure S4. The virulence of phage-resistant PAO1 clones measured in the *G. mellonella* infection model.
