# Supplementary material for: Emerging Phage Resistance in Pseudomonas aeruginosa PAO1 Is Accompanied by an Enhanced Heterogeneity and Reduced Virulence
Source: Viruses. 2021 Jul 10;13(7):1332. doi: 10.3390/v13071332 (PMC8310095; doi:10.3390/v13071332)
Supplement: Supplementary file 1 [file viruses-13-01332-s001.zip › viruses-1278057-Supplementary.pdf]

viruses

MDPI

Article

Emerging Phage Resistance in *Pseudomonas aeruginosa* PAO1 is Accompanied by Enhanced Heterogeneity and Reduced Virulence

Pawel Markwitz [1], Tomasz Olszak [1] , Grzegorz Gula [1], Magdalena Kowalska [2], Michal Arabski [2], and Zuzanna Drulis-Kawa [1,*]

1 Department of Pathogen Biology and Immunology, University of Wroclaw, 51-148 Wroclaw, Poland pawel.markwitz2@uwr.edu.pl (P.M.); tomasz.olszak@uwr.edu.pl (T.O.); grzegorz.gula@uwr.edu.pl (G.G.)
2 Division of Medical Biology, Institute of Biology, Jan Kochanowski University, Poland magdalena.kowalska@ujk.edu.pl (M.K.); arabski@ujk.edu.pl (M.A.)

* Correspondence: zuzanna.drulis-kawa@uwr.edu.pl (Z.D.-K.)

Supplementary materials

A

No. of passages	Lipid A (ng/ml)	normal cells (A-/IP-)	Apoptosis early (A+/IP-)	Apoptosis late (A+/IP+)	Necrosis (A-/IP+)
10	control	99.50 ± 5.08	0.36 ± 5.05	0.10 ± 0.04	0.04 ± 0.03
	100	90.53 ± 4.09	8.47 ± 6.05	0.70 ± 0.08	0.30 ± 0.52
	500	88.70 ± 3.40	5.12 ± 5.31	5.17 ± 2.38	1.01 ± 1.04
15	control	73.93 ± 2.30	18.40 ± 2.45	7.03 ± 2.06	0.63 ± 0.15
	100	90.13 ± 2.25	5.57 ± 0.06	3.60 ± 0.20	0.70 ± 0.14
	500	89.03 ± 0.06	5.40 ± 3.25	4.80 ± 2.25	0.77 ± 0.26

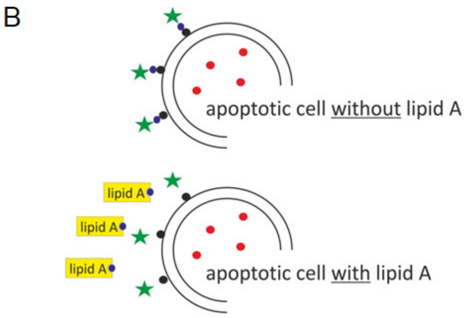

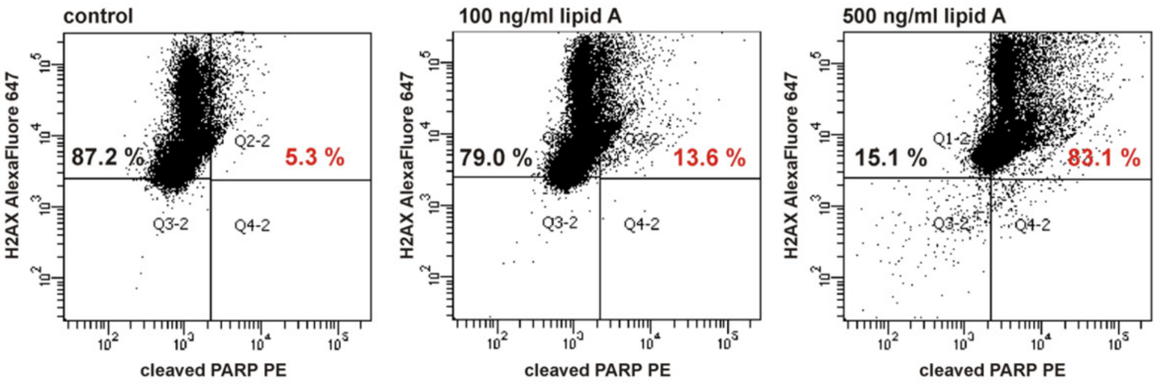

Figure S1. The apoptotic assay measured by Annexin V-FITC apoptosis detection Kit I using *B. cepacia* lipid A as a standard (A) and Cleaved PARP FITC MAB Detection Kit (C). The possible role of lipid A (yellow) in apoptosis detection via, annexin (green star) interactions with phosphatidylserine (black dots in the cell membrane) depending on calcium ions (red dots) is presented (B).

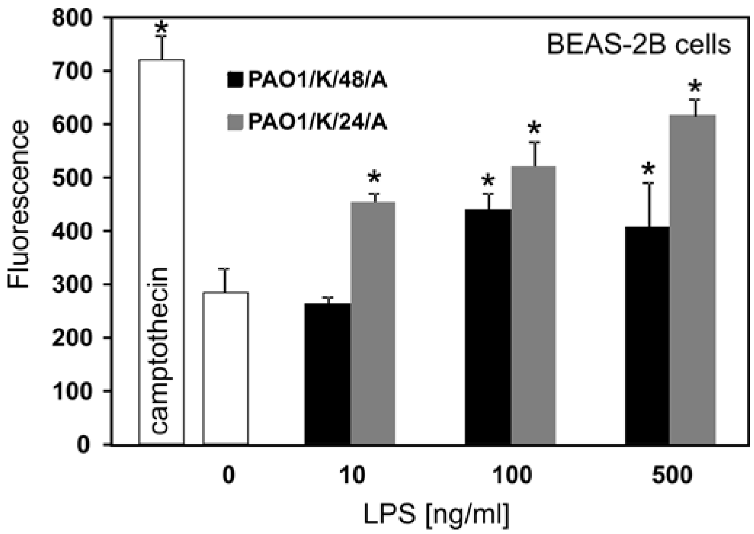

Figure S2. The apoptotic properties of *P. aeruginosa* PAO1 LPS isolated from two clones from the phage-noninfected biofilm. The BEAS-2B cells were treated with LPS at the range of 10-500 ng/ml and the apoptotic effect was measured by Cleaved PARP FITC MAB Detection Kit (C). * $p<0.05$ (ANOVA test).

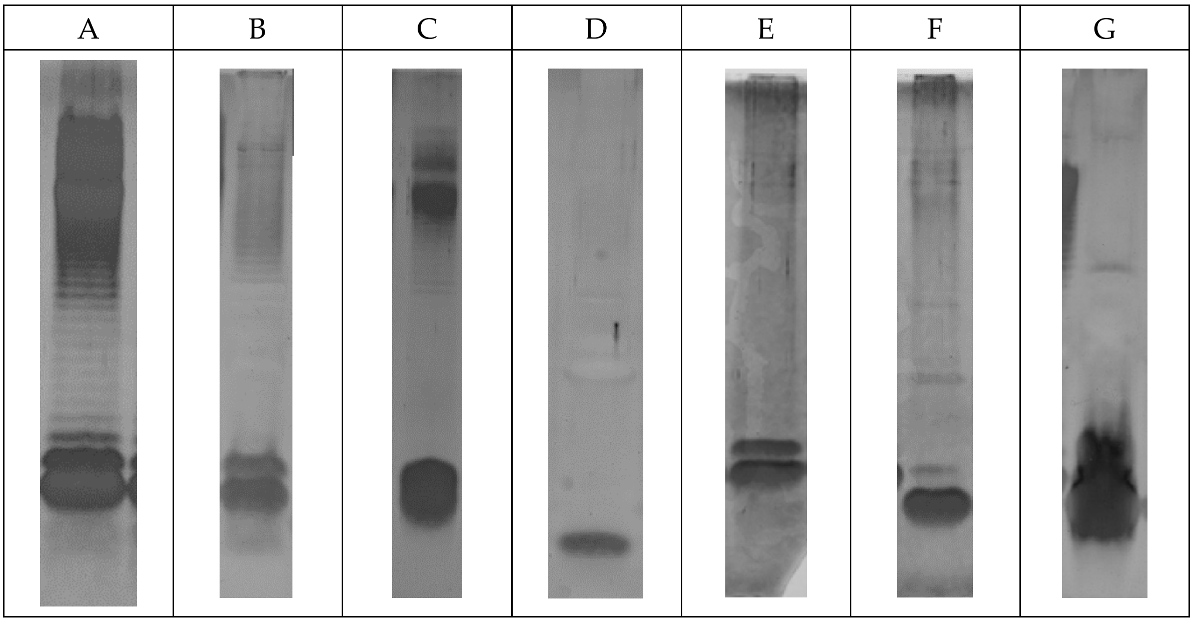

Figure S3. LPS profiles of PAO1 clones isolated after controlled infection by selected phages analyzed in 14% polyacrylamide/tricine-SDS gels: A) smooth profile; B-C) semi-rough profile; D-G) rough profile.

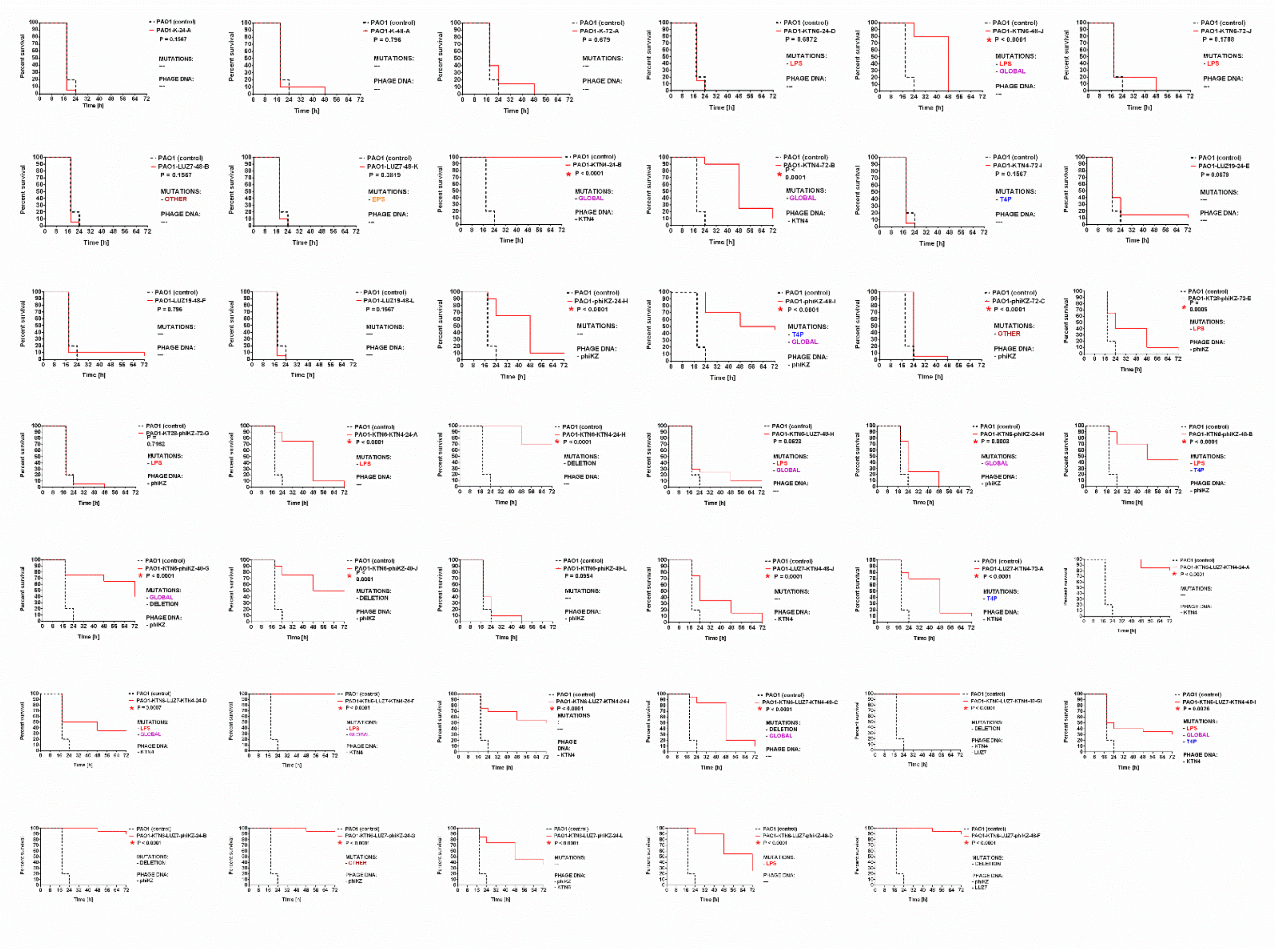

Figure S4. The virulence of phage-resistant PAO1 clones measured in the *G. mellonella* infection model
